# Small cell carcinoma of the prostate presenting with skin metastasis: a case report

**DOI:** 10.1186/1752-1947-8-146

**Published:** 2014-05-12

**Authors:** Kursat Cecen, Mert Ali Karadag, Aslan Demir, Ramazan Kocaaslan

**Affiliations:** 1 Department of Urology, Kafkas University Faculty of Medicine, Kars, Turkey

**Keywords:** Prostatic carcinoma, Skin metastasis, Small cell carcinoma

## Abstract

**Introduction:**

Small cell carcinoma of the prostate is a very rare and aggressive type of prostatic cancer. Most cases are diagnosed at advanced stage due to early metastasis. The bones, liver, regional and distant lymph nodes are the most common sites of metastasis of small cell carcinoma of the prostate. Skin metastasis of small cell carcinoma of the prostate is a very rare entity due to the uncommon metastatic site. Here, we describe the case of a patient with small cell carcinoma of the prostate which metastasized to his skin.

**Case presentation:**

A 74-year-old Caucasian man presented to another urology center for mild lower urinary tract symptoms in 2003. His prostate-specific antigen was 23ng/mL. According to the physical examination signs and prostate-specific antigen, he underwent a transrectal ultrasound-guided prostate biopsy. The pathologic examination of his prostate revealed a Gleason score: 3+4=7 adenocarcinoma of the prostate. Investigations showed stage T2N0M0 disease and he was treated with radiotherapy to his pelvic lymph nodes and prostate. Six years after the initial diagnosis, he complained of a palpable left-side 2×2cm subcutaneous solitary mass localized just behind his scapula. The results of his laboratory tests including serum acid phosphatase and prostate-specific antigen were in normal ranges. Our general surgery department performed a diagnostic biopsy of the mass and totally excised the lesion. The pathologic examination of the mass showed small cell carcinoma metastasis with chromogranin + and the pathologist advised us to examine the lung or prostate for the primary tumor. The patient undertook a transrectal ultrasound-guided prostate biopsy and the pathologic result revealed small cell carcinoma within residual adenocarcinoma. We investigated the other sites for metastasis and restaging investigations showed a 1cm metastatic lesion in his liver. Our medical oncology department decided to treat him with combination chemotherapy with etoposide and cisplatin in six cycles; however, he died due to disseminated myocardial infarction before starting the fifth combination chemotherapy cycle.

**Conclusions:**

Clinicians should keep in mind that early diagnosis of this disease is very difficult due to early metastatic spread of small cell carcinoma and lack of concordant elevation of prostate-specific antigen. There is no accepted standard treatment modality for this pathology and overall prognosis is poor.

## Introduction

The incidence of prostatic carcinoma (PCa) has increased all over the world since the accepted use of prostate-specific antigen (PSA) as a screening tool. In the USA, 192,280 new cases are diagnosed as PCa each year [1]. It is the second leading cause of death among men in the world. Albeit most carcinomas of the prostate are adenocarcinomas, various subtypes of PCa are defined [2]. They have specific clinicopathologic features, clinical relevance and prognosis. These subtypes are squamous cell, sarcomatoid, urothelial, basal cell, adenoid cystic and small cell carcinoma [2].

Small cell carcinoma of the prostate (SCCP) accounts for less than 0.5% to 2% of all malignant PCa and has different clinicopathologic features [2]. SCCP usually accompanies adenocarcinoma of the prostate with a rate of approximately 50% [3]. It is a very aggressive type of cancer and most cases are diagnosed at an advanced stage because of no concordant elevation of PSA [4]. The major symptoms are signs of bladder outlet obstruction in 50% of patients and signs of metastatic disease-like bone pain, neurologic signs, hydronephrosis or abdominal pain in 33% of patients [5]. The bones, liver, regional and distant lymph nodes are the most common sites of metastasis of SCCP [6]. The treatment algorithm of SCCP is a dilemma in urology due to its aggressive nature and uncommon features. It has a very poor response to androgen deprivation therapy. Worldwide accepted treatment modalities are chemotherapy, radiation and surgery [6].

Skin metastasis of SCCP is a very rare entity due to the uncommon metastatic site. Here, we present the case of a patient with SCCP which metastasized to his skin.

## Case presentation

A 74-year-old Caucasian man presented to another urology center for mild lower urinary tract symptoms in 2003. A rectal digital examination of the prostate revealed an enlarged irregular prostate with right-side nodule. Laboratory tests including complete blood count, serum biochemical analysis, urine analysis, and urine culture were performed. All of the results of the tests were normal, except for his PSA value (23ng/mL). In response to the physical examination signs and PSA, he underwent a transrectal ultrasound-guided (TRUSG) prostate biopsy. The pathologic examination of the prostate revealed a Gleason score of 3+4=7 adenocarcinoma of the prostate. After the pathologic result, a computed tomography (CT) of his abdomen with contrast agent and scintigraphy of his bones were performed to decide the stage of the carcinoma. The investigations showed stage T2N0M0 disease. He was diagnosed as localized PCa and treatment alternatives were explained and discussed with him. He preferred radiotherapy and was treated with radiation to his pelvic lymph nodes (46Gy in 23 fractions) and three-dimensional conformal boost of his prostate (24Gy in 12 fractions).

**Figure 1:**
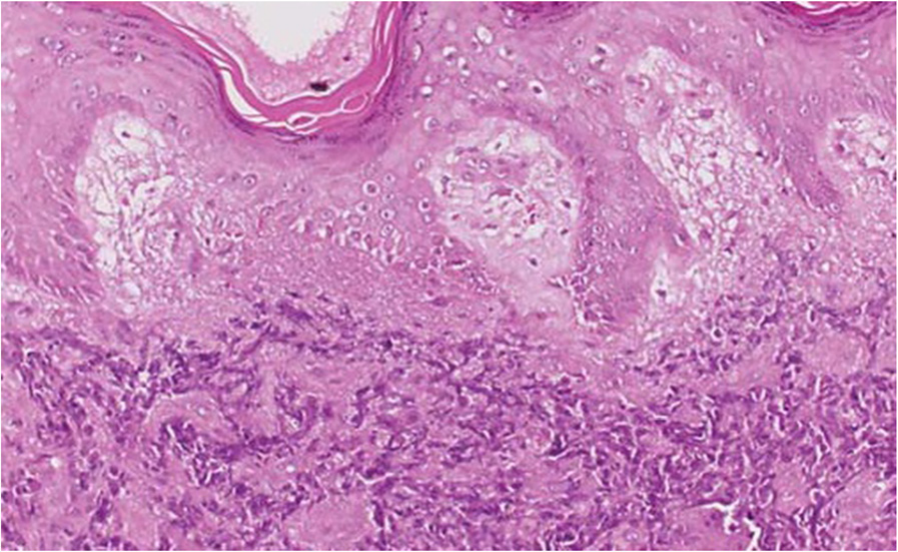
**Microscopic findings of skin metastasis of small cell carcinoma of the prostate.** This figure shows us the microscopic findings of skin metastasis of small cell carcinoma of the prostate in the pathologic examination. It was evaluated after excision of the skin lesion.

**Figure 2:**
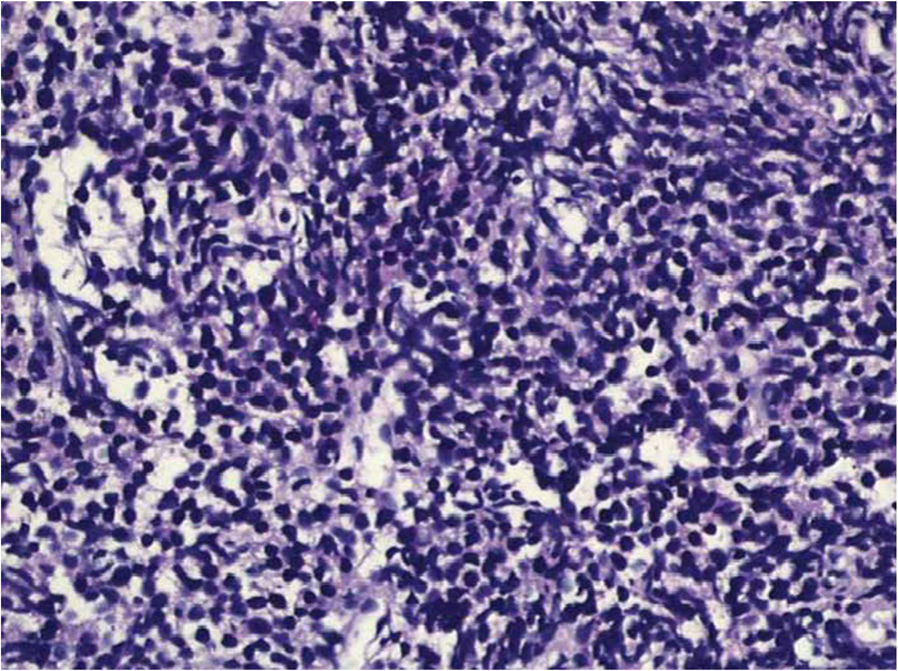
**Small cell carcinoma of the prostate formed by malignant epithelial cells with narrow cytoplasm in the fibromuscular stroma.** This figure shows us the microscopic findings of small cell carcinoma of the prostate in the pathologic examination. The specimen was obtained after a transrectal ultrasound-guided prostate biopsy of the patient.

The recurrence of the carcinoma was not observed in the follow up and he was free of recurrence until 2009. Six years after initial diagnosis and when he was 80-years old, he had a complaint of palpable left-side 2×2cm subcutaneous solitary mass, which localized just behind his scapula. He told us that he had noticed this mass for 2 months. The results of his laboratory tests including serum acid phosphatase and PSA were in normal ranges. The initial diagnosis was considered to be a lipoma and he was referred for a general surgery consultation. The general surgery department performed a diagnostic biopsy of the mass and totally excised the lesion. The pathologic examination of the mass showed small cell carcinoma metastasis with chromogranin + and the pathologist advised us to examine the lung or prostate for the primary tumor (Figure 1). The patient undertook a TRUSG prostate biopsy and the pathologic result revealed small cell carcinoma within residual adenocarcinoma (Figure 2). The Gleason score of the prostatic adenocarcinoma that accompanied small cell carcinoma was 2+2 with an amount of 7%. Like in the metastatic site, the specimen had neuroendocrine differentiation with a chromogranin+. We investigated other sites for metastasis and restaging investigations showed a 1cm metastatic lesion in his liver. His axial skeleton and bones were found to be unusually free of metastasis with nuclear scintigraphy. He consulted our medical oncology department.

Our medical oncology department decided to treat him with combination chemotherapy with etoposide and cisplatin in six cycles. After the fourth cycle, partial response to the treatment was observed and his hepatic lesion was downsized on CT examination; however, he died due to disseminated myocardial infarction before starting the fifth combination chemotherapy cycle.

## Discussion

SCCP is a very rare pathology. It accounts for less than 0.5% to 2% of all malignant PCa [2]. As with adenocarcinoma, this pathology can arise from the peripheral zone of the prostate and can occur without urinary symptoms [7]. The SCCP has an aggressive character and a tendency to metastasize early on to the liver, bones, bladder, rectum, central nervous system, lungs, distant and pelvic lymph nodes and even the pericardium [8]. Skin metastasis is a very uncommon phenomenon and there are only a few reports in the literature about metastasis of SCCP to the skin [9,10]. The disease can be silent and its only manifestation might be symptoms associated with metastasis. For this reason, most patients are diagnosed when SCCP is at an advanced stage. Our patient had no complaints of urinary symptoms at first admission to the hospital or during the follow-up period and diagnosis of the SCCP was maintained only by TRUSG prostate biopsy after pathologic evaluation of the skin lesion. We also noted adenocarcinoma that accompanied SCCP in the second pathologic evaluation of the prostate. The Gleason score of the adenocarcinoma was 2+2 and lower than the initial pathologic result with a Gleason score of 3+4. The difference between the pathologic results may be attributable to the initial radiotherapy treatment.

The clinicopathologic features of SCCP are similar to small cell carcinoma of the lung (SCCL). Like in the lung, vascular invasion, high mitotic index and necroses are common findings. Three theories about its histogenesis have been proposed [7]. The most accepted hypothesis is that prostatic small cell carcinoma arises from totipotential stem cells of the prostate, which can differentiate into either neuroendocrine or epithelial types. Another theory suggests that small cell carcinoma may originate from amine precursor uptake and decarboxylation cells of the endoderm. The last theory depends on the hypothesis that SCCP is a part of the huge spectrum of prostatic adenocarcinomas [7]. The last suggestion is supported by the fact that in 50% of patients SCCP is accompanied by adenocarcinoma of the prostate [3].

Due to histologic similarity to SCCL, paraneoplastic syndromes can be observed in a minority of patients [2]. These can be hyperparathyroidism, thyrotoxicosis, hypercalcemia, hyperglucagonemia and Cushing’s syndrome. Our case did not have signs of any paraneoplastic syndromes. The results of laboratory tests including PSA were in normal ranges during follow up. Elevated serum PSA values can only be observed in patients with SCCP, who have a large amount of adenocarcinoma component. In our case, the PSA value was within normal limits and the amount of adenocarcinoma detected by TRUSG biopsy was very low.

The pathology of neuroendocrine differentiation of small cell carcinoma makes response to androgen deprivation therapy poor. Chromogranin A, a neuroendocrine marker, might be elevated if there is a neuroendocrine differentiation of the carcinoma [2]. Berruti *et al*. suggested that the presence of chromogranin in newly diagnosed PCa was an independent predictive factor of hormone refractory disease in patients under androgen deprivation therapy and associated with diminished overall survival [11]. This marker is known to activate androgen receptors, even in the absence of androgens. Also, the pathologic examination of our patient revealed positivity for chromogranin. If we used androgen deprivation therapy in our case instead of chemotherapy, the patient would probably have had a poor response to therapy due to the presence of chromogranin.

The mean survival of patients with SCCP ranges between 5 and 17 months and less than 5% of cases survive beyond 24 months [5]. The treatment algorithm of SCCP is a dilemma in urology due to its rarity, aggressive nature and uncommon features. The treatment of SCCP is similar to that of patients who have SCCL. As chemotherapeutic agents, cyclophosphamide, etoposide, doxorubicin, vincristine and cisplatin with or without doxorubicin might be used for treating patients with SCCP [9,10]. Other agents like carboplatin, gemcitabine and docetaxel have also been investigated in studies and showed benefits with acceptable tolerable adverse effects [12]. We preferred six cycles of combination chemotherapy with etoposide and cisplatin for our patient according to the advice of our medical oncology department; but the treatment period was unfinished because the patient died after the fourth cycle. However, four cycles of the chemotherapy regimen in our patient downsized the metastatic foci in his liver.

The other treatment modalities of SCCP are surgery and radiation therapy. In the literature, there is only one case with SCCP reported to be cured after radical prostatectomy [12]. However, Papandreou *et al*. suggested that surgery could not be the proper treatment modality of SCCP due to the fact that most cases with SCCP have a distant metastasis at initial diagnosis [13]. Radiation therapy might be considered for local control of the disease with chemotherapy [14]. In our case, we did not prefer radiotherapy due to metastatic foci in the patient’s liver. Chemotherapy was thought to be the proper treatment alternative for our patient.

## Conclusions

Small cell carcinoma can accompany prostatic adenocarcinoma in 50% of patients and should be suspected when the metastatic spread of PCa is unusual for adenocarcinoma. Clinicians should keep in mind that early diagnosis of this disease is very difficult due to early metastatic spread of small cell carcinoma and lack of concordant elevation of PSA. There is no accepted standard treatment modality for this pathology and overall prognosis is poor.

## Consent

Written consent was obtained from the patient’s next-of-kin for publication of this case report and accompanying images. A copy of the written consent is available for review by the Editor-in-Chief of this journal.

## Abbreviations

CT: Computed tomography; PCa: Prostatic carcinoma; PSA: Prostate-specific antigen; SCCL: Small cell carcinoma of the lung; SCCP: Small cell carcinoma of the prostate; TRUSG: Transrectal ultrasound-guided

## Competing interests

The authors declare that they have no competing interests.

## Authors’ contributions

KC diagnosed, treated and maintained follow up of the patient. MAK collected the data and wrote the manuscript. AD photographed the patient and performed consultations with the pathology department. RK maintained follow up of the patient. All authors read and approved the final manuscript.

## Authors’ information

KC is assistant professor of urology and working as head of the urology department. MAK, AD, and RK are assistant professors of urology.
